# ZFAS1: A novel vital oncogenic lncRNA in multiple human cancers

**DOI:** 10.1111/cpr.12513

**Published:** 2018-10-05

**Authors:** Anbang He, Shiming He, Xuesong Li, Liqun Zhou

**Affiliations:** ^1^ Department of Urology The Institute of Urology Peking University First Hospital Peking University National Urological Cancer Centre Beijing 100034 China

**Keywords:** cancer, lncRNA, review, ZFAS1

## Abstract

Long noncoding RNAs (lncRNAs) are a class of noncoding, endogenous, single‐stranded RNAs longer than 200 nucleotides in length that are transcribed by RNA polymerase II. Mounting evidence has indicated that lncRNAs play key roles in several physiological and pathological processes by modifying gene expression at the transcriptional, posttranscriptional, epigenetic, and translation levels. Many reports have demonstrated that lncRNAs function as potential oncogene or tumour suppressors and thus play vital regulatory roles in tumourigenesis and tumour progression. ZNFX1 antisense RNA 1 (ZFAS1), a novel lncRNA transcribed in the antisense orientation of zinc finger NFX1‐type containing 1(ZNFX1), was found to be increased in multiple cancers, such as gastric cancer and hepatocellular carcinoma, contributing to cancer development and progression. In the present review, we summarized recent progression on study of the functions and underlying molecular mechanisms of ZFAS1 related to occurrence and development of multiple cancers.

## 1 INTRODUCTION

Recently, due to increasing incidence and mortality, cancer is one of the leading public health problems all over the world and continues to capture our great attention.^1^ In the light of estimates in a comprehensive overview called Cancer Statistics, 2017, there were 1 688 780 new patients predicted to be diagnosed with cancers and 600 920 patients were predicted to die of cancers in the United States are predicted to occur in 2017.^2^ In China, there were an estimated 4 292 000 new cancer patients and 2 814 000 patients who died of cancers have occurred in 2015.^3^ Despite multiple treatments modalities being accessible for cancer patients, including surgery, chemotherapy, radiation therapy, and targeted therapy, the 3‐ and 5‐year cancer‐specific survival rates still remain poor.^1^, ^4^ Exploration of and revealing the molecular mechanisms important to cancer is therefore critical in order to identify new diagnostic and prognostic biomarkers as well as effective treatments.

Less than 2% of genes are associated with the production of specific proteins according to eukaryote whole‐genome sequencing.^5^ Furthermore, more than 70% of identified genes had no protein‐coding function.^5^ At first, such noncoding RNAs (ncRNAs) were considered to be transcriptional noise with no specific biological function.^6^ With further research of ncRNAs, however, they have been identified as indispensable regulators of a variety of biological processes, including epigenetics, cell cycle, posttranscriptional regulation and chromatin modification.^6^, ^7^, ^8^ Although we currently lack satisfactory classifications for these ncRNAs, long ncRNAs are arbitrarily considered to be longer than 200 nucleotides.^6^, ^9^ Mounting evidence has indicated that lncRNA plays key roles in physiological and pathological processes by modifying gene expression at the transcriptional, posttranscriptional, epigenetic, and translation levels.^6^, ^7^, ^8^ Several reports have demonstrated that lncRNAs could function as potential oncogenes or tumour suppressor genes in order to play vital regulatory roles in tumourigenesis and tumour progression.^10^, ^11^

**Figure 1:**
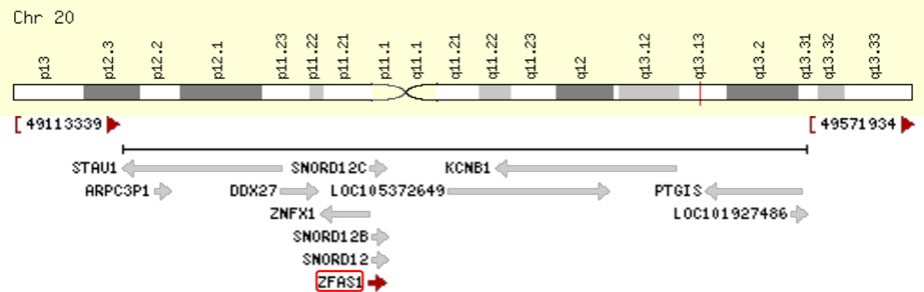
ZFAS1 was located on chromosome 20q13.13 and transcribed in antisense orientation of ZNFX1 and also hosts three small nucleolar RNAs, including Snord12, Snord12b, and Snord12c

ZNFX1 antisense RNA 1 (ZFAS1), a novel lncRNA transcribed from the antisense orientation of zinc finger NFX1‐type containing 1(ZNFX1), is located on chromosome 20q13.13 (Figure 1).^12^ Recent research has reported that ZFAS1 may act an emerging regulatory factor in multiple diseases, such as acute myocardial infarction,^13^, ^14^ rheumatoid arthritis,^15^ and cancer.^16^ Accumulating evidence has suggested that the abnormal expression of ZFAS1 contributes to the occurrence and development of various cancers, including gastric cancer, hepatocellular carcinoma, colorectal cancer, glioma, osteosarcoma, ovarian cancer, acute myeloid leukaemia, nonsmall cell lung cancer (NSCLC), oesophageal squamous cell carcinoma, and breast cancer. In this review, we summarized recent progression regarding study of the functions and underlying mechanisms of ZFAS1 in the occurrence and development of various cancers.

## 2 DISCOVERY of ZFAS1

ZFAS1, firstly studied in breast cancer by Marjan, is transcribed from the gene ZNFX1 on the antisense DNA strand near the 5′ end. It is located in both cellular cytoplasmic and nuclear fractions with a length of 17 561 bp, including five exons with five transcriptional variations (NR_003604.3:1008 bp; NR_003605.2:689 bp; NR_003606.3:860 bp; NR_036658.2: 946 bp; NR_036659.2:504 bp). ZFAS1 hosts three small nucleolar RNAs (snoRNAs), including Snord12, Snord12b, and Snord12c.^12^ SnoRNAs, a class of RNA molecules with 60‐150 nt in length which may be involved in the chemical modifications of other RNAs, like transfer RNAs, ribosomal RNAs, and small nuclear RNAs.^17^ Current research has indicated that upregulation of ZFAS1 is positively correlated with clinicopathological features and prognosis, including TNM stage, lymph‐node metastasis, and overall survival. Several meta‐analyses have indicated that lncRNA ZFAS1 could be as a prognostic biomarker for patients with various cancers.^16^, ^18^, ^19^, ^20^ Upregulated ZFAS1 could function as an oncogene to promoting cell proliferation, migration, and invasion. The relevant clinicopathological features and underlying molecular mechanisms of ZFAS1 in various cancers are summarized in Tables 1 and 2.

**Table 1:** Functional characterizations of ZFAS1 in multiple human cancers

Cancer types	Expression	Role	Functional role	Related genes	References
Colorectal cancer	Upregulated	Oncogenic	Cell proliferation migration, invasive, metastasis, cell cycle control, cell antiapoptosis	miR‐484 miR‐590‐3p p53 and EMT signalling pathway	21, 22, 23, 24
Gastric cancer	Upregulated	Oncogenic	Cell proliferation, cell antiapoptosis, cell migration and metastasis, cell cycle control	EMT, KLF2, EZH2, NKD2	25, 26, 27
Hepatocellular carcinoma	Upregulated	Oncogenic	Cell invasion and tumour metastasis	miR‐150, ZEB1, MMP14, and MMP16	28
Glioma	Upregulated	Oncogenic	Cell proliferation, migration, and invasive	Notch and EMT signalling pathway	30, 31
Osteosarcoma	Upregulated	Oncogenic	Cell proliferation, migration and invasion, cell antiapoptosis, cell cycle control	miR‐200b/c, miR‐486, BMI1, ZEB2	32, 33
Ovarian cancer	Upregulated	Oncogenic	Cell proliferation migration, chemoresistance	miR‐150‐5p, Sp1	34, 35
Acute myeloid leukaemia	Upregulated	Oncogenic	Cell proliferation, cell antiapoptosis, cell cycle control		36
Nonsmall cell lung cancer	Upregulated	Oncogenic			37
Oesophageal squamous cell carcinoma	Upregulated	Oncogenic			29

**Table 2:** Clinical features of ZFAS1 in multiple human cancers

Cancer types	Clinicopathological features	References
Colorectal cancer	Positive *helicobacter pylori*, advanced TNM stage, positive lymph‐node metastasis, poorer overall survival, higher recurrence rate, positive lymphatic invasion, and positive microvascular invasion	21, 22, 23, 24
Gastric cancer	Advanced TNM stage, higher T stage, positive lymph‐node metastasis, larger tumour size	25, 26, 27
Hepatocellular Carcinoma	Positive microvascular invasion, shorter overall survival, and higher recurrence rate	28
Oesophageal squamous cell carcinoma	Poorer histological grade, shorter overall survival	29
Glioma	Higher clinical stage, poorer overall survival	30, 31
Osteosarcoma	Poorer overall survival	32, 33
Ovarian cancer	Poorer overall survival, advanced tumour stage, larger tumour size, positive lymph‐node metastasis	34, 35
Nonsmall cell lung cancer	Advanced TNM stage, positive lymph‐node metastasis, poor differentiation, poorer overall survival	37

## 3 ZFAS1 IN VARIOUS HUMAN CANCERS

Accumulating evidence has demonstrated the abnormal expression of ZFAS1 in multiple cancers, including gastric cancer, hepatocellular carcinoma, colorectal cancer, glioma, osteosarcoma, ovarian cancer, acute myeloid leukaemia, NSCLC, oesophageal squamous cell carcinoma, and breast cancer. Specifically, the expression of ZFAS1 was found to be upregulated in most cancers, except breast cancer. Upregulation of ZFAS1 expression was positively associated with advanced TNM stage and lymph‐node metastasis in colorectal cancer, gastric cancer, ovarian cancer, and NSCLC. Additionally, increased expression of ZFAS1 was closely correlated with poorer overall survival in colorectal cancer, gastric cancer, glioma, osteosarcoma, oesophageal squamous cell carcinoma, ovarian cancer, and NSCLC. The association between ZFAS1 expression and the relevant clinicopathological features of various cancers are summarized in Table 1. Overexpression of ZFAS1 was shown to promote cell proliferation and induced cellular apoptosis in various cancers, including colorectal cancer, gastric cancer, glioma, osteosarcoma, acute myeloid leukaemia, and ovarian cancer. Interestingly, knockdown of ZFAS1 inhibited cell migration and invasion in colorectal cancer, gastric cancer, glioma, hepatocellular carcinoma, osteosarcoma. The functions and underlying molecular mechanisms of ZFAS1 in various cancers are summarized in Table 2 and detailed in the rest of this review.

### 3.1 Colorectal cancer

ZFAS1 has been found in several studies to be overexpressed in colorectal cancer tissues and cell lines compared with paired adjacent normal colorectal tissues and a human colonic epithelial cell line, HCoEpiC. Fang et al^21^ demonstrated that upregulated ZFAS1 expression was positively associated with an advanced TNM stage, vascular invasion, and lymph‐node metastasis. Overexpression of ZFAS1 promoted cell proliferation, invasion, and induced cell apoptosis in colorectal cancer (CRC). Furthermore, silencing of ZFAS1 could reduce Zinc Finger E‐Box Binding Homeobox 1 (ZEB1) expression and increased the expression of epithelial markers, including E‐cadherin and ZO‐1, meanwhile decreasing the expression of mesenchymal markers, including vimentin and N‐cadherin. Taking into account these results, the researchers concluded that ZFAS1 could function as an oncogene by promoting ZEB1 induction of epithelial‐to‐mesenchymal transition (EMT).

Wang et al^22^ confirmed that the expression of ZFAS1 was higher in CRC tissues than in adjacent normal colorectal tissues. Moreover, compared with primary CRC tumours, ZFAS1 was upregulated in metastatic tumours, suggesting ZFAS1 may have a specific role in such cancer metastasis. Furthermore, ZFAS1 expression in CRC was positively correlated with lymphatic invasion and TNM stage. Increased ZFAS1 levels were determined to predict poor overall survival and short relapse‐free survival and cox multivariate analyses revealed that ZFAS1 expression is an independent prognostic factor in CRC. Silencing ZFAS1 expression could suppress cell migration, invasive, and metastasis ability of CRC cell in vitro and in vivo. Xie et al^23^ also confirmed that elevated ZFAS1 expression was significantly associated with advanced TNM stage, lymph‐nodes metastasis, and poor overall survival. Functional experiments indicated that downregulated ZFAS1 inhibited cell proliferation and invasion both in vitro and in vivo. Bioinformatics analysis and experiments also proved that ZFAS1 directly interacted with miR‐484. Thorenoor et al^24^ showed that silencing ZFAS1 decreased cyclin‐dependent kinase 1 (CDK1) to inhibit cell proliferation through G1‐arrest of cell cycle by sponging miR‐590‐3p in CRC cells. Decreased ZFAS1 expression resulted in reduction of p53 and cyclin B1 levels and promoting poly ADP‐ribose polymerase (PARP) cleavage to induce cell apoptosis. These findings demonstrated that ZFAS1 could act an oncogenic lncRNA which may serve as a novel independent prognostic biomarker for patients with CRC.

### 3.2 Gastric cancer

Current research trends have explored the function and molecular mechanisms of ZFAS1in gastric cancer. Zhou et al,^25^ Pan et al,^26^ and Nie et al^27^ all demonstrated the elevated expression of ZFAS1 in gastric cancer tissues and cell lines, compared to adjacent nontumour tissues and normal gastric cell line (GES‐1). Increased expression of ZFAS1 in gastric cancer was also closely interrelated with TNM stage, lymph‐node metastasis, and tumour size. Kaplan‐Meier analysis revealed that GC patients with higher ZFAS1 expression levels had poorer overall survival (OS) and progression‐free survival (PFS) than those with lower ZFAS1 expression levels. Knockdown of ZFAS1 suppressed cancer cells proliferation and promoted cancer cells apoptosis in vitro, and restrained tumourigenesis of gastric cancer cells in vivo. Further research showed that ZFAS1 could simultaneously interact with the enhancer of zeste homolog 2 (EZH2) and lysine demethylase 1A/histone demethyltransferase of REST complex (LSD1/CoREST) to inhibit Kruppel Like Factor 2 (KLF2) and Naked Cuticle Homolog 2 (NKD2) transcription. Moreover, rescue experiments suggested that ZFAS1 partially relied on suppression of KLF2 and NKD2 to exert its oncogenic effects. Zhou et al^25^ found that circulating ZFAS1 was also overexpressed in GC patients and that surgery operation could decrease subsequent expression in plasma. Finally, to elucidate the potential diagnostic value of plasma ZFAS1, receiver operating characteristic (ROC) curve and the Youden index were calculated. The area under the ROC curve (AUC) was 0.727 (95% CI: 0.642‐0.813, *P* < 0.001), with a sensitivity of 0.766 and specificity of 0.639. All of these results indicated that circulating ZFAS1 had a relatively moderate accuracy when distinguishing gastric cancer patients from other individuals. Pan et al^26^ also found that silencing of ZFAS1 repressed cell proliferation and migration by inhibiting cell cycle and EMT. Finally, ZFAS1 was observed in exosomes and was transported by exosomes to promote cell proliferation and migration of GC.

### 3.3 Hepatocellular carcinoma

Li et al^28^ reported that ZFAS1 expression was significantly amplified in HCC tissues and cell lines compared with the paired nontumour tissues and normal liver cells. Correlation analysis demonstrated that high ZFAS1 levels were positively associated with microvascular invasion and recurrence. Kaplan‐Meier analysis suggested that HCC patients with upregulated ZFAS1 experienced poorer overall survival (OS) and progression‐free survival (PFS). Further study showed that amplified ZFAS1 could enhance HCC cell invasion and tumour metastasis in vitro and in vivo. Subsequent bioinformatics analysis and experimentation revealed that ZFAS1 directly interacted with miR‐150. Moreover, miR‐150 could suppress HCC cell invasion and metastasis by targeting ZEB1, matrix metallopeptidase 14 (MMP14), and matrix metallopeptidase 16 (MMP16). From these results, the authors concluded that ZFAS1 was acting in an oncogenic role by binding miR‐150 and abrogating its tumour‐suppressive function in HCC progression.

### 3.4 Oesophageal squamous cell carcinoma

Shi et al^29^ demonstrated that ZFAS1 expression was elevated in oesophageal squamous cell carcinoma (ESCC) and high ZFAS1 was positively correlated with histological grade. ESCC patients with high ZFAS1 expression had poorer OS than those with low ZFAS1 expression both in the primary cohort and validation cohort. In both cohorts, both univariate and multivariate analysis confirmed that ZFAS1 expression, histological grade, and *T* stage as prognostic factors. A nomogram then used clinicopathological factors and ZFAS1 expression to predict the prognosis of lymph‐node‐negative ESCC patients without preoperative chemoradiotherapy. The resulting decision‐making curve suggested that the proposed nomogram was better than 8th AJCC‐TNM staging system in terms of survival prediction.

### 3.5 Glioma

ZFAS1 expression was markedly overexpressed in glioma tissues and cell lines in reports by Gao et al^30^ and Lv et al^31^ with high ZFAS1 expression in glioma tissues being closely associated with advanced clinical stage and poor overall survival. Both univariate and multivariable Cox regression analysis revealed that upregulated ZFAS1 and the clinical stage were independent prognostic factors in the overall survival of glioma patients. Silencing of ZFAS1 could suppress cell proliferation by arresting the cell cycle and inducing cell apoptosis in glioma. Furthermore, knockdown ZFAS1 inhibited cell migration and invasion in glioma. Several EMT markers play indispensable roles in epithelial‐mesenchymal transition signalling pathway, including MMP2, MMP9, E‐cadherin, N‐cadherin, Integrin β1, ZEB1, Twist, and Snail. After silencing ZFAS1, the expression levels of MMP2, MMP9, N‐cadherin, Integrin β1, ZEB1, Twist, and Snail were markedly downregulated, while E‐cadherin expression was significantly upregulated. Furthermore, the expression of Notch signal‐related proteins Hes family BHLH transcription factor 1 (Hes‐1) and NICD were decreased by silencing ZFAS1. Notch signalling pathway is believed to function as a vital regulator of embryo development via the regulation of intercellular signal communication by modifying cell proliferation and apoptosis. Therefore, these data indicated that ZFAS1 may enhance glioma progression by activating the EMT and Notch signalling pathways.

### 3.6 Osteosarcoma

In osteosarcoma tissues and cell lines, Liu et al and Li et al^32^, ^33^ showed that ZFAS1 was markedly upregulated. Kaplan‐Meier analysis suggested that osteosarcoma patients with high ZFAS1 levels had shorter overall survival (OS) than those with low ZFAS1. Functional experiments revealed that upregulated ZFAS1 promoted osteosarcoma cell proliferation, migration, and invasion in vitro and in vivo. Mechanistically, the researchers discovered that ZFAS1 regulated malignant phenotypes by competitively binding the miR‐200b/c to increase B Lymphoma Mo‐MLV Insertion Region 1 Homolog (BMI1) expression. Further RNA pull‐down assays proved that ZFAS1 interacted with ZEB2 to keep ZEB2 protein stability. Moreover, they found that specificity protein 1 (SP1) acted as an upstream activated factor of ZFAS1. Li et al discovered that ZFAS1 sponges miR‐486 to promote osteosarcoma cells progression and metastasis in vitro and vivo.

### 3.7 Ovarian cancer

A report by Xia et al^34^ showed that ZFAS1 was upregulated in epithelial ovarian cancer (EOC) tissues and cell lines. Amplified expression ZFAS1 in EOC tissues was positively correlated with advanced clinical stage, larger tumour size, and lymph‐node metastasis. Kaplan‐Meier analysis suggested that EOC patients with high ZFAS1 expression had poorer overall survival (OS) than those with low ZFAS1. Cell experiments revealed that knockdown of ZFAS1 suppressed cell proliferation, migration, and chemoresistance in EOC. Further research identified downregulated miR‐150‐5p as a potential target of ZFAS1 in EOC tissue. Further luciferase assays indicated miR‐150‐5p subsequently suppressed transcription factor SP1 expression. Subsequent rescue experiments revealed than silencing miR‐150‐5p could partially rescue the suppressed proliferation and migration induced by inhibition of ZFAS1 in EOC cells. These data suggested that ZFAS1/miR‐150‐5p/SP1 axis may play a critical role in enhancing cell proliferation, migration, and chemoresistance in EOC. Additionally, Liu et al^35^ identified eight lncRNA signatures, which were remarkably correlated with chemosensitivity in a multivariate logistic regression model and could precisely predict the chemosensitivity of patients, by analysing the lncRNA expression profiles of 258 HGS‐OvCa patients from The Cancer Genome Atlas(TCGA). Similarly, data from the Gene Expression Omnibus datasets (GEO) confirmed the strong relation between ZFAS1 and chemosensitivity. In vitro experiments suggested that the ZFAS1 expression was overexpressed with cisplatin treatment in several EOC cell lines (A2008, HeyA8, and HeyC2). Taken together, these findings suggested that ZFAS1 may play a vital role in platinum resistance, which should be further explored.

### 3.8 Acute myeloid leukaemia

When compared with normal cell lines, Guo et al^30^ revealed that the expression of ZFAS1 was increased in all four human acute myeloid leukaemia (AML) cell lines. The silencing of ZFAS1 suppressed cell proliferation in HL‐60 and SKNO‐1 cell lines according to CCK‐8 assay and induced AML cell cycle G1 phase arrest and promoted cell apoptosis by flow cytometry. These findings demonstrated that upregulated ZFAS1 enhanced cell proliferation and suppressed cell apoptosis in AML.

### 3.9 Nonsmall cell lung cancer

Tian et al^36^ demonstrated that the expression of ZFAS1 was increased in nonsmall cell lung cancer (NSCLC) compared with adjacent noncancerous tissues. Again, increased ZFAS1 was positively correlated with advanced TMN stage, lymph‐node metastasis, and poor differentiation. Kaplan‐Meier analysis indicated that NSCLC patients with upregulated ZFAS1 had poorer OS than those with downregulated ZFAS1. Both univariate and multivariable Cox regression analysis suggested that high ZFAS1 expression TMN stage, lymph‐node metastasis, and differentiation level were independent prognostic factors for overall survival of NSCLC patients.

### 3.10 Breast cancer

Marjan et al^12^ identified that ZFAS1 as highly expressed in stage of lactation in mouse mammary gland tissues and was reduced in human invasive ductal breast carcinoma compared with normal breast tissues. On the contrary, Hansji et al^37^ showed that ZFAS1 expression was not significantly different in breast cancer tissues from normal tissues according to RNAseq data set (HiSeqV2‐2015‐02‐24) from TCGA. However, ZFAS1 expression was significantly downregulated in basal and HER2 breast cancer subtypes compared to normal breast tissue. Importantly, ER+ breast tumours had higher expression of ZFAS1 than ER− (negative) breast tumours. These two studies had different results largely due to their limited number of samples and testing of different breast cancer subtypes. Next, there was no significant found between the expression level of ZFAS1 and ZNFX1. They also found that ZFAS1 was localized to both the cytoplasm and the nucleus while ZNFX1 was enriched in the nucleus. Further research has indicated that ZFAS1 is associated with only part of the ribosomes, being enriched in 18S rRNA fractions compared to those containing 28S rRNA. The expression of ZFAS1 is associated with the expression of genes which were involved in ribosome biogenesis. A series of experiments have also demonstrated that ZFAS1 is induced upon ribosome biogenesis, revealing a role in the synthesis or assembly of ribosomes.

## 4 UNDERLYING MOLECULAR MECHANISMS OF ZFAS1

### 4.1 The molecular level

#### 4.1.1 Function of ZFAS1 as a ceRNA

**Figure 2:**
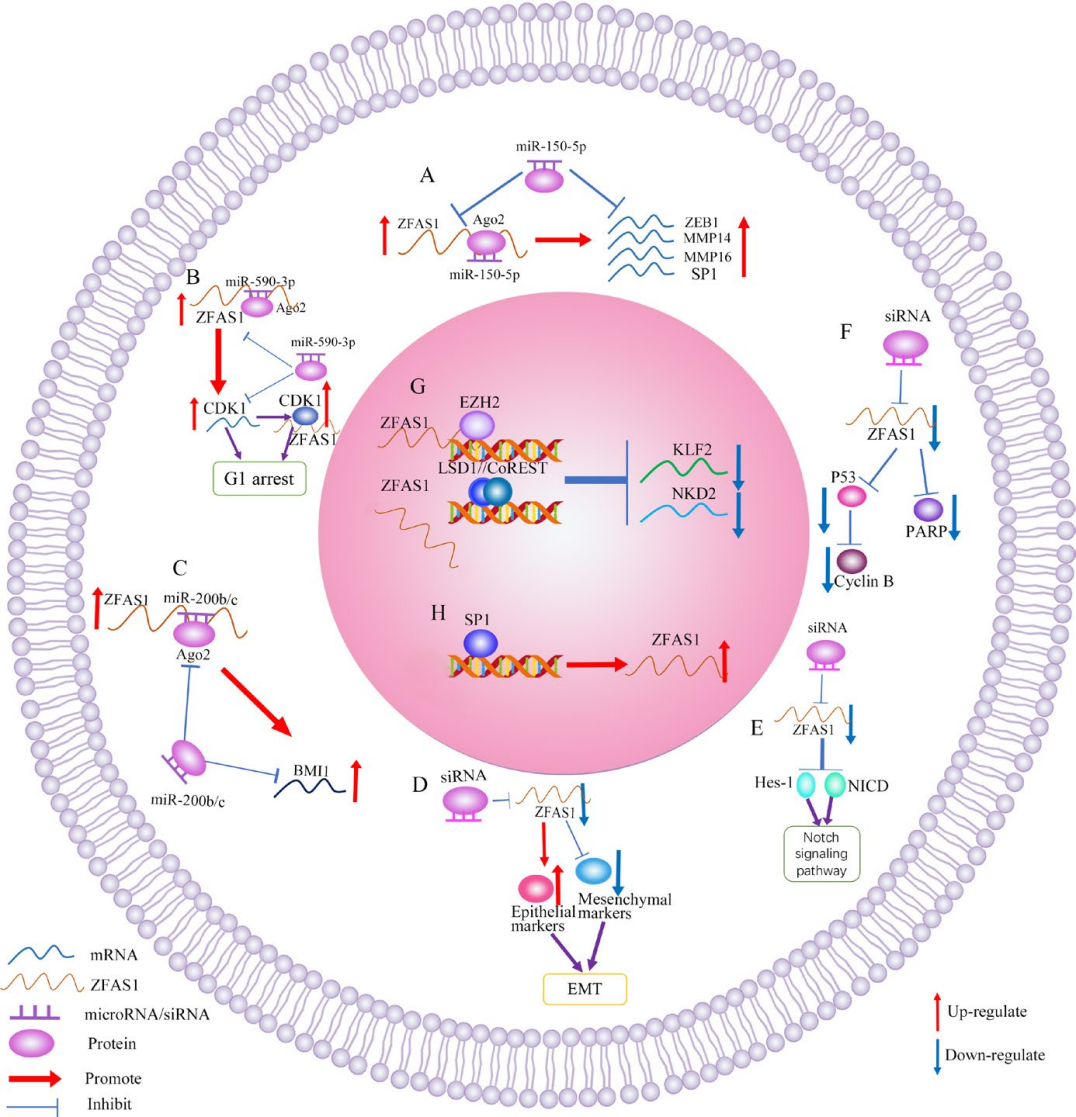
Underlying molecular mechanisms of ZFAS1 in multiple human cancers. A, ZFAS1 functioned as a ceRNA to directly interacted with miR‐150 to increase the expression of ZEB1, MMP14, MMP16, and SP1. B, silencing ZFAS1 could decrease the expression of CDK1 by competing for miR‐590‐3p. C, ZFAS1 may act as a ceRNA to upregulate the expression of BMI1 by competitively binding miR‐200b/c. D, After Silencing ZFAS1, the expression levels of mesenchymal markers, including MMP2, MMP9, N‐cadherin, Integrin β1, ZEB1, Twist, and Snail, were markedly downregulated, while the expression of epithelial markers including E‐cadherin and ZO1 significantly upregulated. E, ZFAS1 could regulate the expression of Hes‐1 and NICD to activate Notch signalling pathway. F, decreased ZFAS1 contributed to cell‐cycle arrest and inducement of apoptosis by reducing the expression of p53 and cyclin B1 and promoting PARP cleavage. G, ZFAS1 could simultaneously interact with EZH2 and LSD1/CoREST to inhibit KLF2 and NKD2 transcription. H, SP1 could activate the expression of ZFAS1

Competing endogenous RNAs (ceRNAs), a class of noncoding RNA, have been shown to modify the mRNA expression of genes by acting as miRNA sponges competing for mutual microRNAs, which simultaneously target both noncoding RNA and genes.^38^, ^39^ Several articles have demonstrated that ZFAS1 could function as a ceRNA to regulate the expression of specific genes by competing for specific microRNAs, exerting its oncogenic function in various cancers. Li et al and Xia et al showed that ZFAS1 directly interacted with miR‐150 to increase the expression of ZEB1, MMP14, MMP16, and transcription factor SP1 (Figure 2A). Thorenoor et al then showed that silencing of ZFAS1 could decrease the expression of CDK1 in order to inhibit cell proliferation via G1 arrest of the cell cycle by competing for miR‐590‐3p in CRC cells (Figure 2B). Liu et al found that ZFAS1 promoted malignant phenotypes by competitively binding miR‐200b/c to upregulate the expression of BMI1 (Figure 2C). The ring finger protein encoded by BMI1 is major component of PRC1, which acts as an indispensable epigenetic repressor of a variety of regulatory genes involved in self‐renewal and embryonic development by remodelling the chromatin of stem cells. Finally, Xie et al proved that ZFAS1 could directly interact with miR‐484. Previous study identified miR‐484 as an inhibitor of cell proliferation and invasion by targeting ZEB1 and SMAD2 in cervical cancer cells, revealing that ZFAS1 may promote cell proliferation and invasion via sponging miR‐484 to modulate the expression of ZEB1 and SMAD2.

#### 4.1.2 Transcriptional regulation

Further research has demonstrated that ZFAS1 could simultaneously interact with EZH2 and LSD1/CoREST to inhibit KLF2 and NKD2 transcription. Rescue experiments revealed that ZFAS1 was partly relied on suppressing KLF2 and NKD2 expression to exert its oncogenic effects (Figure 2G). SP1 was also demonstrated to be an upstream factor for the activation of ZFAS1 expression (Figure 2H). Moreover, Hansji et al showed that ZFAS1 was induced upon ribosome biogenesis, revealing a role in synthesis or assembly of ribosomes.

### 4.2 ZFAS1 is involved in several signalling pathways

#### 4.2.1 EMT signalling pathway

EMT plays an essential role in both physiological and pathological processes, such as embryonic development as well as the occurrence and development of tumours.^40^, ^41^ Several EMT markers are indispensable in the EMT signalling pathway, including MMP2, MMP9, E‐cadherin, N‐cadherin, Integrin β1, ZEB1/2, Twist, and Snail.^41^ After silencing of ZFAS1, the expression levels of MMP2, MMP9, N‐cadherin, Integrin β1, ZEB1, Twist, and Snail were all markedly downregulated, while E‐cadherin expression significantly upregulated. In conclusion, Liu et al also showed that ZFAS1 interacted with ZEB2 to protect ZEB2 protein stability in the activation of the EMT signalling pathway.

#### 4.2.2 Notch signalling pathway

The Notch signalling pathway functions as a vital regulator in embryo development via the regulation of intercellular signal communication by modifying cell proliferation and apoptosis^42^, ^43^, ^44^; both Hes‐1 and NICD have vital functions in Notch signalling pathway.^44^, ^45^ Gao et al found that the expression Notch signal‐related proteins Hes‐1 and NICD were decreased by silencing ZFAS1in glioma cell, suggesting ZFAS1 could regulate the expression of Hes‐1 and NICD to activate Notch signalling pathway (Figure 2E).

#### 4.2.3 p53 signalling pathway

The p53 protein, a nuclear transcription factor, modified the expression of multiple genes which are involved in a variety of biological processes, including the cell cycle, apoptosis, and DNA repair.^46^ Thorenoor et al demonstrated that decreased ZFAS1 contributed to cell‐cycle arrest and induction of apoptosis by reducing the expression of p53 and cyclin B1 and promoting PARP cleavage (Figure 2F). This finding suggests that ZFAS1 activated the p53 signalling pathway to act oncogenic roles in various cancers.

## 5 CONCLUSION AND FUTURE PERSPECTIVES

With the rapid development of next‐generation sequencing technology, a large amount of evidence has identified dysregulated lncRNAs as potential oncogenes or tumour suppressor genes that play crucial regulatory roles in tumourigenesis and tumour progression.^47^, ^48^ ZFAS1, a newly identified lncRNA, was found to be widely upregulated in various human cancers (expect breast cancer). Amplified ZFAS1 was dramatically correlated with multiple clinicopathological features and prognosis, such as TNM stage, lymph‐node metastasis, and overall survival. In vitro, knockdown of ZFAS1 repressed cell proliferation, invasion, and promoted cell apoptosis in multiple cancers, suggesting ZFAS1 contributed to tumourigenesis and tumour progression. In vivo experiments also confirmed that silencing ZFAS1 could obviously restrain tumourigenesis of cancer cells. The underlying molecular mechanisms of ZFAS1 involved in multiple cancers have been explored preliminarily. At the molecular level, ZFAS1 could function as a ceRNA in the regulation of specific gene expression by competing for specific microRNAs, representing its oncogenic function in various cancers. ZFAS1 also could interact with some proteins to regulate gene expression at the transcriptional level. Furthermore, ZFAS1 was demonstrated to take part in several signalling pathways contributing to carcinogenesis and cancer progression, including EMT, Notch, and p53 signalling pathways.

Although some of the underlying mechanisms of ZFAS1 involved in different cancers have been identified, there are many problems that still need to be addressed. First, the molecular mechanism of ZFAS1 in each cancer type should be clarified. Many lncRNAs function at the epigenetic, transcriptional, and posttranslational processing level in their contribution to carcinogenesis and cancer progression. Second, finding a diagnostic biomarker or therapeutic target is a promising direction for cancer diagnosis and treatment. Recently, several lncRNAs have been identified not only in neoplastic tissues but also in body fluids, such as plasma and urine, suggesting a potential role as cancer‐specific molecular biomarkers in cancer diagnosis, prognosis, and treatment. Other advantages in cancer diagnosis and treatment, especially in the early diagnosis and prediction of prognosis, include simple and noninvasive detection as well as a moderate treatment price. Therefore, the clinical value of ZFAS1 in the diagnosis and treatment of cancers requires more attention.

In summary, ZFAS1 has been shown to have oncogenic function in tumourigenesis and tumour progression and may act as a potential cancer‐specific molecular biomarker in the general diagnosis, prognosis, and treatment of cancer. Until this point, research on the mechanism of ZFAS1 has made some progression, but remains in the early stages. Future works will need to emphasize exploration of the precise molecular regulatory mechanisms of ZFAS1 in carcinogenesis and cancer progression, to facilitate the clinical application of ZFAS1 as early as possible.

## AUTHORS’ CONTRIBUTIONS

AH, SH wrote the manuscript. XL and LZ provided the financial support and reviewed the manuscript. All authors read and approved the final manuscript.

## CONFLICT OF INTEREST

All authors declare no conflict of interest.
